# 4-Chloro-2-(6-nitro-1*H*-benzimidazol-2-yl)phenol *N*,*N*-dimethyl­form­amide solvate

**DOI:** 10.1107/S1600536810041243

**Published:** 2010-10-20

**Authors:** Abeer Mohamed Farag, Siang Guan Teoh, Hasnah Osman, Chin Sing Yeap, Hoong-Kun Fun

**Affiliations:** a School of Chemical Sciences, Universiti Sains Malaysia, 11800 USM, Penang, Malaysia; b X-ray Crystallography Unit, School of Physics, Universiti Sains Malaysia, 11800 USM, Penang, Malaysia

## Abstract

In the title compound, C_13_H_8_ClN_3_O_3_·C_3_H_7_NO, the benzimidazole and benzene rings make a dihedral angle of 0.63 (11)°. An intra­molecular O—H⋯N hydrogen bond generates an *S*(6) ring motif. The solvent mol­ecule is hydrogen-bonded to the benzimidazole mol­ecule by inter­molecular N—H⋯O and C—H⋯O hydrogen bonds, generating an *R* ^1^_2_(7) ring motif. In the crystal, the mol­ecules are arranged into parallel layers perpendicular to the *c* axis and stabilized by weak π–π inter­actions [centroid–centroid distances in the range 3.4036 (18)–3.5878 (16) Å].

## Related literature

For general background to and the biological activity of benzimidazole derivatives, see: Trivedi *et al.* (2006 ▶); White *et al.* (2004 ▶); Garuti *et al.* (2004 ▶). For related structures, see: Eltayeb *et al.* (2009 ▶); Yeap *et al.* (2009 ▶). For the stability of the temperature controller used in the data collection, see: Cosier & Glazer (1986 ▶). For hydrogen-bond motifs, see: Bernstein *et al.* (1995 ▶).
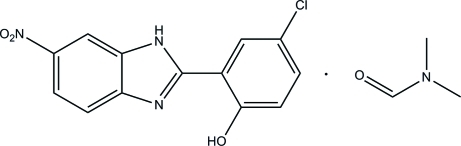


## Experimental

### 

#### Crystal data

C_13_H_8_ClN_3_O_3_·C_3_H_7_NO
*M* *_r_* = 362.77
Monoclinic, 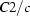

*a* = 15.200 (2) Å
*b* = 18.355 (2) Å
*c* = 13.279 (3) Å
β = 119.232 (2)°
*V* = 3233.1 (9) Å^3^
*Z* = 8
Mo *K*α radiation
μ = 0.27 mm^−1^
*T* = 100 K
0.45 × 0.12 × 0.05 mm

#### Data collection

Bruker APEXII DUO CCD area-detector diffractometer
Absorption correction: multi-scan (*SADABS*; Bruker, 2009 ▶) *T*_min_ = 0.889, *T*_max_ = 0.987
14542 measured reflections
3719 independent reflections
2771 reflections with *I* > 2σ(*I*)
*R*_int_ = 0.041

#### Refinement

*R*[*F* ^2^ > 2σ(*F* ^2^)] = 0.065
*wR*(*F* ^2^) = 0.201
*S* = 1.07
3719 reflections
228 parameters
H-atom parameters constrained
Δρ_max_ = 0.78 e Å^−3^
Δρ_min_ = −0.34 e Å^−3^

### 

Data collection: *APEX2* (Bruker, 2009 ▶); cell refinement: *SAINT* (Bruker, 2009 ▶); data reduction: *SAINT*; program(s) used to solve structure: *SHELXTL* (Sheldrick, 2008 ▶); program(s) used to refine structure: *SHELXTL*; molecular graphics: *SHELXTL*; software used to prepare material for publication: *SHELXTL* and *PLATON* (Spek, 2009 ▶).

## Supplementary Material

Crystal structure: contains datablocks global, I. DOI: 10.1107/S1600536810041243/ng5046sup1.cif

Structure factors: contains datablocks I. DOI: 10.1107/S1600536810041243/ng5046Isup2.hkl

Additional supplementary materials:  crystallographic information; 3D view; checkCIF report

## Figures and Tables

**Table 1:** Hydrogen-bond geometry (Å, °)

*D*—H⋯*A*	*D*—H	H⋯*A*	*D*⋯*A*	*D*—H⋯*A*
O3—H1*O*3⋯N2	0.75	1.90	2.568 (4)	149
N1—H1*N*1⋯O4	0.89	1.86	2.736 (4)	167
C13—H13*A*⋯O4	0.93	2.58	3.472 (5)	161

**Fig. 1.**
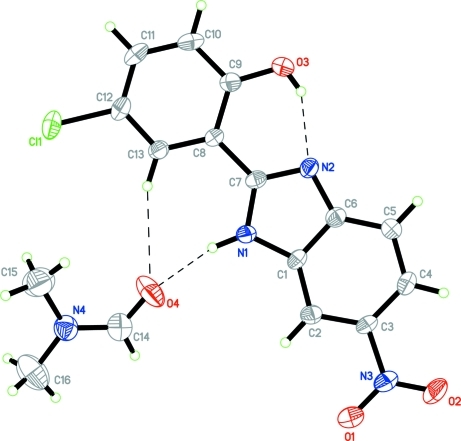
The molecular structure of title compound with atom labels and 50% probability ellipsoids for non-H atoms. Intramolecular hydrogen bonds are shown as dashed lines.

**Fig. 2.**
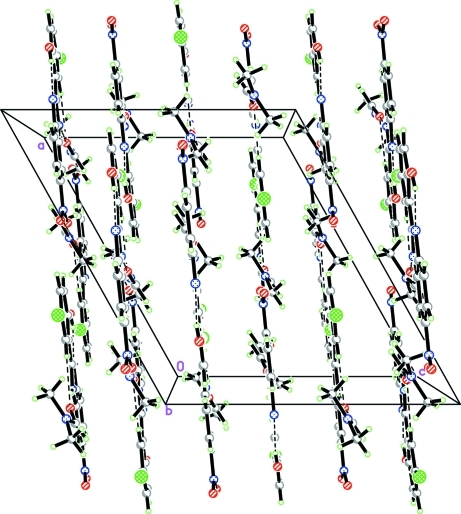
The crystal packing of title compound viewed down b axis, showing the molecules are arranged into parallel layers.

## supplementary crystallographic information

### Comment

Benzimidazole and its derivatives are widely used in biological systems (Trivedi *et al.*, 2006). Some derivatives of benzimidazole are used as inhibitors of the DNA-repair enzyme poly (ADP-ribose) polymerase-1 (PARP-1) (White *et al.*, 2004) and antiproliferative activities (Garuti *et al.*, 2004). In view of the biological importance of aforementioned benzimidazole, the crystal structure determination of the title compound was carried out and the result is presented here.

The asymmetric unit of title compound consists of one benzimidazole molecule and one dimethylformamide solvent (Fig. 1). The geometric parameters are comparable to those related structures (Eltayeb *et al.*, 2009; Yeap *et al.*, 2009). The molecular structure of the benzimidazole is essentially planar with the maximum deviation of 0.071 Å for atom O1. An intramolecular O3—H1O3···N2 hydrogen bond generate *S*(6) ring motif (Bernstein *et al.*, 1995). The solvent molecule is hydrogen-bonded to the benzimidazole molecule by intermolecular N1—H1N1···O4 and C13—H13A···O4 hydrogen bonds generating *R*^1^_2_(7) ring motif. In the crystal packing, the molecules are arranged into parallel layers perpendicular to *c* axis and stabilized by weak π···π interactions [*Cg*1···*Cg*1^i^ of 3.4036 (18) Å, *Cg*1···*Cg*1^ii^ of 3.5247 (17) Å and *Cg*1···*Cg*2^i^ of 3.5878 (16) Å; (i) -*x*, *y*, 1/2 - *z*; (ii) -*x*, 1 - *y*, 1 - *z. Cg*1 and *Cg*2 are centroids of N1–C1–C6–N2–C7 and C1–C6 rings, respectively].

### Experimental

5-Chloro-2-hydroxy benzaldehyde (0.626 g, 4 mmol) was added to the solution of 4-nitrobenzene-1,2-diamine (0.306 g, 2 mmol) in methanol (30 ml). The mixture was refluxed with stirring for 1 h. The resultant solid obtained was then filtered and washed with methanol. Orange plate-shaped single crystals of the title compound suitable for X-ray structure determination was obtained from DMF by slow evaporation at room temperature.

### Refinement

The O– and N-bound hydrogen atoms were located from difference Fourier map and refined using a riding model [*U*_iso_(H) = 1.2*U*_eq_ (N) or 1.5*U*_eq_(O)]. The rest of hydrogen atoms were positioned geometrically [C–H = 0.93 & 0.96 Å] and refined using a riding model [*U*_iso_(H) = 1.2 & 1.5*U*_eq_(C)]. A rotating-group model were applied for methyl groups.

### Crystal data

**Table:**

C_13_H_8_ClN_3_O_3_·C_3_H_7_NO	*F*(000) = 1504
*M**_r_* = 362.77	*D*_x_ = 1.491 Mg m^−^^3^
Monoclinic, *C*2/*c*	Mo *K*α radiation, λ = 0.71073 Å
Hall symbol: -C 2yc	Cell parameters from 3050 reflections
*a* = 15.200 (2) Å	θ = 2.8–29.6°
*b* = 18.355 (2) Å	µ = 0.27 mm^−^^1^
*c* = 13.279 (3) Å	*T* = 100 K
β = 119.232 (2)°	Plate, orange
*V* = 3233.1 (9) Å^3^	0.45 × 0.12 × 0.05 mm
*Z* = 8	

### Data collection

**Table:**

Bruker APEXII DUO CCD area-detector diffractometer	3719 independent reflections
Radiation source: fine-focus sealed tube	2771 reflections with *I* > 2σ(*I*)
graphite	*R*_int_ = 0.041
φ and ω scans	θ_max_ = 27.5°, θ_min_ = 1.9°
Absorption correction: multi-scan (*SADABS*; Bruker, 2009)	*h* = −19→19
*T*_min_ = 0.889, *T*_max_ = 0.987	*k* = −23→23
14542 measured reflections	*l* = −17→17

### Refinement

**Table:**

Refinement on *F*^2^	Primary atom site location: structure-invariant direct methods
Least-squares matrix: full	Secondary atom site location: difference Fourier map
*R*[*F*^2^ > 2σ(*F*^2^)] = 0.065	Hydrogen site location: inferred from neighbouring sites
*wR*(*F*^2^) = 0.201	H-atom parameters constrained
*S* = 1.07	*w* = 1/[σ^2^(*F*_o_^2^) + (0.0961*P*)^2^ + 7.3038*P*] where *P* = (*F*_o_^2^ + 2*F*_c_^2^)/3
3719 reflections	(Δ/σ)_max_ < 0.001
228 parameters	Δρ_max_ = 0.78 e Å^−^^3^
0 restraints	Δρ_min_ = −0.34 e Å^−^^3^

### Special details

**Table:**

Experimental. The crystal was placed in the cold stream of an Oxford Cryosystems Cobra open-flow nitrogen cryostat (Cosier & Glazer, 1986) operating at 100.0 (1) K.
Geometry. All e.s.d.'s (except the e.s.d. in the dihedral angle between two l.s. planes) are estimated using the full covariance matrix. The cell e.s.d.'s are taken into account individually in the estimation of e.s.d.'s in distances, angles and torsion angles; correlations between e.s.d.'s in cell parameters are only used when they are defined by crystal symmetry. An approximate (isotropic) treatment of cell e.s.d.'s is used for estimating e.s.d.'s involving l.s. planes.
Refinement. Refinement of *F*^2^ against ALL reflections. The weighted *R*-factor *wR* and goodness of fit *S* are based on *F*^2^, conventional *R*-factors *R* are based on *F*, with *F* set to zero for negative *F*^2^. The threshold expression of *F*^2^ > σ(*F*^2^) is used only for calculating *R*-factors(gt) *etc*. and is not relevant to the choice of reflections for refinement. *R*-factors based on *F*^2^ are statistically about twice as large as those based on *F*, and *R*- factors based on ALL data will be even larger.

### Fractional atomic coordinates and isotropic or equivalent isotropic displacement parameters (Å^2^)

**Table:**

	*x*	*y*	*z*	*U*_iso_*/*U*_eq_	
Cl1	−0.29053 (6)	0.25697 (4)	0.23736 (8)	0.0465 (3)	
O1	0.37235 (16)	0.53844 (13)	0.5412 (2)	0.0418 (6)	
O2	0.36636 (17)	0.65517 (14)	0.5483 (2)	0.0444 (6)	
O3	−0.28130 (16)	0.57579 (11)	0.23880 (19)	0.0362 (5)	
H1O3	−0.2293	0.5904	0.2591	0.054*	
N1	−0.00189 (16)	0.46895 (13)	0.37354 (18)	0.0242 (5)	
H1N1	0.0057	0.4209	0.3772	0.029*	
N2	−0.08820 (17)	0.57321 (12)	0.33366 (19)	0.0245 (5)	
N3	0.32588 (18)	0.59601 (15)	0.5240 (2)	0.0327 (6)	
C1	0.06745 (19)	0.52535 (15)	0.4062 (2)	0.0227 (5)	
C2	0.1718 (2)	0.52401 (16)	0.4543 (2)	0.0261 (6)	
H2A	0.2085	0.4809	0.4719	0.031*	
C3	0.21629 (19)	0.59173 (15)	0.4736 (2)	0.0250 (6)	
C4	0.1634 (2)	0.65760 (16)	0.4484 (2)	0.0304 (6)	
H4A	0.1978	0.7016	0.4629	0.036*	
C5	0.0598 (2)	0.65720 (16)	0.4018 (2)	0.0287 (6)	
H5A	0.0233	0.7004	0.3852	0.034*	
C6	0.0120 (2)	0.59004 (15)	0.3807 (2)	0.0250 (6)	
C7	−0.09359 (19)	0.50080 (15)	0.3299 (2)	0.0228 (5)	
C8	−0.18949 (19)	0.46199 (14)	0.2832 (2)	0.0218 (5)	
C9	−0.2793 (2)	0.50277 (16)	0.2409 (2)	0.0269 (6)	
C10	−0.3707 (2)	0.46580 (18)	0.2000 (3)	0.0342 (7)	
H10A	−0.4303	0.4923	0.1719	0.041*	
C11	−0.3743 (2)	0.39127 (18)	0.2004 (2)	0.0339 (7)	
H11A	−0.4355	0.3674	0.1750	0.041*	
C12	−0.2861 (2)	0.35177 (17)	0.2390 (2)	0.0301 (6)	
C13	−0.1943 (2)	0.38598 (15)	0.2804 (2)	0.0254 (6)	
H13A	−0.1357	0.3586	0.3064	0.030*	
O4	0.0524 (2)	0.32533 (15)	0.3936 (3)	0.0728 (9)	
N4	0.0760 (2)	0.20375 (15)	0.4053 (2)	0.0374 (6)	
C14	0.0997 (3)	0.2715 (2)	0.3954 (4)	0.0526 (9)	
H14A	0.1575	0.2787	0.3892	0.063*	
C15	−0.0141 (3)	0.1904 (3)	0.4121 (4)	0.0611 (11)	
H15A	−0.0317	0.2334	0.4394	0.092*	
H15B	−0.0026	0.1509	0.4645	0.092*	
H15C	−0.0681	0.1779	0.3370	0.092*	
C16	0.1378 (4)	0.1427 (2)	0.4137 (4)	0.0710 (13)	
H16A	0.1942	0.1590	0.4061	0.106*	
H16B	0.0989	0.1085	0.3534	0.106*	
H16C	0.1616	0.1195	0.4873	0.106*	

### Atomic displacement parameters (Å^2^)

**Table:**

	*U*^11^	*U*^22^	*U*^33^	*U*^12^	*U*^13^	*U*^23^
Cl1	0.0443 (5)	0.0298 (4)	0.0618 (5)	−0.0138 (3)	0.0230 (4)	−0.0003 (3)
O1	0.0249 (11)	0.0443 (14)	0.0522 (13)	−0.0012 (10)	0.0157 (10)	−0.0013 (10)
O2	0.0298 (12)	0.0452 (14)	0.0561 (14)	−0.0158 (10)	0.0192 (10)	−0.0078 (11)
O3	0.0252 (10)	0.0291 (11)	0.0515 (13)	0.0043 (8)	0.0164 (10)	−0.0016 (9)
N1	0.0197 (11)	0.0250 (12)	0.0278 (11)	0.0006 (9)	0.0116 (9)	0.0019 (9)
N2	0.0230 (11)	0.0238 (12)	0.0279 (11)	−0.0039 (9)	0.0135 (9)	−0.0012 (9)
N3	0.0229 (12)	0.0402 (15)	0.0341 (12)	−0.0047 (11)	0.0133 (10)	−0.0033 (10)
C1	0.0209 (12)	0.0270 (14)	0.0217 (11)	−0.0059 (10)	0.0115 (10)	−0.0023 (10)
C2	0.0226 (13)	0.0301 (15)	0.0271 (12)	0.0008 (11)	0.0134 (10)	−0.0006 (10)
C3	0.0172 (12)	0.0326 (15)	0.0248 (12)	−0.0051 (10)	0.0101 (10)	−0.0024 (10)
C4	0.0292 (15)	0.0276 (15)	0.0334 (14)	−0.0087 (11)	0.0145 (11)	−0.0046 (11)
C5	0.0273 (14)	0.0251 (14)	0.0325 (14)	0.0016 (11)	0.0138 (11)	−0.0001 (11)
C6	0.0235 (13)	0.0276 (14)	0.0235 (12)	−0.0018 (11)	0.0112 (10)	−0.0020 (10)
C7	0.0196 (12)	0.0290 (14)	0.0217 (11)	−0.0008 (10)	0.0114 (10)	0.0009 (10)
C8	0.0187 (12)	0.0248 (14)	0.0219 (11)	−0.0025 (10)	0.0100 (10)	−0.0007 (9)
C9	0.0226 (13)	0.0294 (15)	0.0281 (13)	0.0011 (11)	0.0119 (11)	0.0008 (10)
C10	0.0186 (13)	0.0455 (19)	0.0371 (15)	0.0020 (12)	0.0124 (12)	−0.0009 (13)
C11	0.0227 (14)	0.0443 (18)	0.0328 (14)	−0.0118 (12)	0.0121 (11)	−0.0024 (12)
C12	0.0288 (14)	0.0309 (15)	0.0299 (13)	−0.0094 (12)	0.0138 (11)	−0.0005 (11)
C13	0.0213 (13)	0.0276 (14)	0.0264 (12)	−0.0011 (10)	0.0110 (10)	0.0019 (10)
O4	0.073 (2)	0.0341 (15)	0.099 (2)	0.0211 (14)	0.0322 (18)	0.0026 (14)
N4	0.0405 (15)	0.0347 (15)	0.0394 (14)	−0.0003 (11)	0.0214 (12)	−0.0028 (11)
C14	0.048 (2)	0.041 (2)	0.066 (2)	−0.0015 (17)	0.0258 (18)	0.0046 (17)
C15	0.048 (2)	0.070 (3)	0.064 (2)	−0.001 (2)	0.027 (2)	0.009 (2)
C16	0.092 (4)	0.051 (3)	0.078 (3)	0.023 (2)	0.049 (3)	0.000 (2)

### Geometric parameters (Å, °)

**Table:**

Cl1—C12	1.741 (3)	C8—C13	1.397 (4)
O1—N3	1.229 (3)	C8—C9	1.411 (4)
O2—N3	1.212 (3)	C9—C10	1.396 (4)
O3—C9	1.341 (3)	C10—C11	1.369 (5)
O3—H1O3	0.7498	C10—H10A	0.9300
N1—C7	1.354 (3)	C11—C12	1.384 (4)
N1—C1	1.387 (3)	C11—H11A	0.9300
N1—H1N1	0.8875	C12—C13	1.376 (4)
N2—C7	1.331 (4)	C13—H13A	0.9300
N2—C6	1.370 (3)	O4—C14	1.216 (5)
N3—C3	1.463 (3)	N4—C14	1.319 (5)
C1—C2	1.391 (4)	N4—C16	1.431 (5)
C1—C6	1.399 (4)	N4—C15	1.437 (5)
C2—C3	1.378 (4)	C14—H14A	0.9300
C2—H2A	0.9300	C15—H15A	0.9600
C3—C4	1.399 (4)	C15—H15B	0.9600
C4—C5	1.383 (4)	C15—H15C	0.9600
C4—H4A	0.9300	C16—H16A	0.9600
C5—C6	1.388 (4)	C16—H16B	0.9600
C5—H5A	0.9300	C16—H16C	0.9600
C7—C8	1.461 (4)		
			
C9—O3—H1O3	110.0	O3—C9—C8	123.1 (2)
C7—N1—C1	106.1 (2)	C10—C9—C8	118.9 (3)
C7—N1—H1N1	122.1	C11—C10—C9	121.2 (3)
C1—N1—H1N1	131.8	C11—C10—H10A	119.4
C7—N2—C6	106.1 (2)	C9—C10—H10A	119.4
O2—N3—O1	123.4 (3)	C10—C11—C12	119.5 (3)
O2—N3—C3	119.1 (3)	C10—C11—H11A	120.3
O1—N3—C3	117.5 (2)	C12—C11—H11A	120.3
N1—C1—C2	130.7 (3)	C13—C12—C11	121.3 (3)
N1—C1—C6	106.4 (2)	C13—C12—Cl1	119.1 (2)
C2—C1—C6	122.9 (3)	C11—C12—Cl1	119.6 (2)
C3—C2—C1	114.6 (3)	C12—C13—C8	119.7 (3)
C3—C2—H2A	122.7	C12—C13—H13A	120.1
C1—C2—H2A	122.7	C8—C13—H13A	120.1
C2—C3—C4	124.2 (3)	C14—N4—C16	123.0 (4)
C2—C3—N3	118.6 (3)	C14—N4—C15	118.9 (3)
C4—C3—N3	117.1 (2)	C16—N4—C15	118.0 (4)
C5—C4—C3	119.9 (3)	O4—C14—N4	125.4 (4)
C5—C4—H4A	120.0	O4—C14—H14A	117.3
C3—C4—H4A	120.0	N4—C14—H14A	117.3
C4—C5—C6	117.6 (3)	N4—C15—H15A	109.5
C4—C5—H5A	121.2	N4—C15—H15B	109.5
C6—C5—H5A	121.2	H15A—C15—H15B	109.5
N2—C6—C5	130.4 (3)	N4—C15—H15C	109.5
N2—C6—C1	108.9 (2)	H15A—C15—H15C	109.5
C5—C6—C1	120.8 (2)	H15B—C15—H15C	109.5
N2—C7—N1	112.6 (2)	N4—C16—H16A	109.5
N2—C7—C8	122.2 (2)	N4—C16—H16B	109.5
N1—C7—C8	125.2 (2)	H16A—C16—H16B	109.5
C13—C8—C9	119.4 (2)	N4—C16—H16C	109.5
C13—C8—C7	121.8 (2)	H16A—C16—H16C	109.5
C9—C8—C7	118.8 (2)	H16B—C16—H16C	109.5
O3—C9—C10	118.0 (3)		
			
C7—N1—C1—C2	−179.0 (3)	C6—N2—C7—C8	−179.2 (2)
C7—N1—C1—C6	0.6 (3)	C1—N1—C7—N2	−0.9 (3)
N1—C1—C2—C3	178.8 (2)	C1—N1—C7—C8	179.1 (2)
C6—C1—C2—C3	−0.8 (4)	N2—C7—C8—C13	−179.5 (2)
C1—C2—C3—C4	0.2 (4)	N1—C7—C8—C13	0.4 (4)
C1—C2—C3—N3	−180.0 (2)	N2—C7—C8—C9	−0.2 (4)
O2—N3—C3—C2	−175.2 (3)	N1—C7—C8—C9	179.8 (2)
O1—N3—C3—C2	3.6 (4)	C13—C8—C9—O3	−178.9 (2)
O2—N3—C3—C4	4.6 (4)	C7—C8—C9—O3	1.7 (4)
O1—N3—C3—C4	−176.6 (2)	C13—C8—C9—C10	1.7 (4)
C2—C3—C4—C5	0.5 (4)	C7—C8—C9—C10	−177.7 (2)
N3—C3—C4—C5	−179.3 (2)	O3—C9—C10—C11	−179.4 (3)
C3—C4—C5—C6	−0.7 (4)	C8—C9—C10—C11	0.0 (4)
C7—N2—C6—C5	178.3 (3)	C9—C10—C11—C12	−1.9 (4)
C7—N2—C6—C1	−0.4 (3)	C10—C11—C12—C13	2.1 (4)
C4—C5—C6—N2	−178.5 (3)	C10—C11—C12—Cl1	−178.9 (2)
C4—C5—C6—C1	0.1 (4)	C11—C12—C13—C8	−0.4 (4)
N1—C1—C6—N2	−0.1 (3)	Cl1—C12—C13—C8	−179.4 (2)
C2—C1—C6—N2	179.5 (2)	C9—C8—C13—C12	−1.5 (4)
N1—C1—C6—C5	−179.0 (2)	C7—C8—C13—C12	177.9 (2)
C2—C1—C6—C5	0.6 (4)	C16—N4—C14—O4	−176.2 (4)
C6—N2—C7—N1	0.8 (3)	C15—N4—C14—O4	1.6 (6)

### Hydrogen-bond geometry (Å, °)

**Table:**

*D*—H···*A*	*D*—H	H···*A*	*D*···*A*	*D*—H···*A*
O3—H1O3···N2	0.75	1.90	2.568 (4)	149
N1—H1N1···O4	0.89	1.86	2.736 (4)	167
C13—H13A···O4	0.93	2.58	3.472 (5)	161

## References

[bb1] Bernstein, J., Davis, R. E., Shimoni, L. & Chang, N.-L. (1995). *Angew. Chem. Int. Ed. Engl.* **34**, 1555–1573.

[bb2] Bruker (2009). *APEX2*, *SAINT* and *SADABS* Bruker AXS Inc., Madison, Wisconsin, USA.

[bb3] Cosier, J. & Glazer, A. M. (1986). *J. Appl. Cryst.* **19**, 105–107.

[bb4] Eltayeb, N. E., Teoh, S. G., Fun, H.-K., Jebas, S. R. & Adnan, R. (2009). *Acta Cryst.* E**65**, o1374–o1375. 10.1107/S1600536809018698 PMC2969736 21583223

[bb5] Garuti, L., Roberti, M., Pizzirani, D., Pession, A., Leoncini, E., Cenci, V. & Hrelia, S. (2004). *Farmaco*, **59**, 663–668. 10.1016/j.farmac.2004.04.001 15262537

[bb6] Sheldrick, G. M. (2008). *Acta Cryst.* A**64**, 112–122. 10.1107/S0108767307043930 18156677

[bb7] Spek, A. L. (2009). *Acta Cryst.* D**65**, 148–155. 10.1107/S090744490804362X PMC2631630 19171970

[bb8] Trivedi, R., De, S. K. & Gibbs, R. A. (2006). *J. Mol. Catal. A*, **245**, 8–11.

[bb9] White, A., Curtin, N. J., Eastman, B., Golding, B. T., Hostomsky, Z., Kyle, S., Li, J., Maegley, K., Skalitzky, D., Webber, S., Yu, X. & Griffin, R. J. (2004). *Biorg* *Med. Chem. Lett.* **14**, 2433–2437. 10.1016/j.bmcl.2004.03.017 15109627

[bb10] Yeap, C. S., Kargar, H., Kia, R., Jamshidvand, A. & Fun, H.-K. (2009). *Acta Cryst.* E**65**, o745–o746. 10.1107/S1600536809008071 PMC2969036 21582478

